# Unveiling Complexity: Black Pleural Effusion Due to a Pancreaticopleural Fistula

**DOI:** 10.7759/cureus.58687

**Published:** 2024-04-21

**Authors:** Vladimir Aleksiev, Boyko Yavorov, Daniel Markov, Filip Shterev, Desislav Stanchev, Bozhidar Hristov, Ilia Todorov

**Affiliations:** 1 Department of Cardiovascular Surgery, Medical University of Plovdiv, Plovdiv, BGR; 2 Department of General and Clinical Pathology, Medical University of Plovdiv, Plovdiv, BGR; 3 Section Pneumonology and Physiatrics, Department of Internal Diseases, Medical University of Plovdiv, Plovdiv, BGR; 4 Section Gastroenterology, Department of Internal Diseases, Medical University of Plovdiv, Plovdiv, BGR

**Keywords:** black pleural effusion, pancreaticopleural fistula, pancreatic pseudocyst, thoracocentesis, hydrothorax

## Abstract

Black pleural effusions (BPEs) are an exceedingly rare class of exudative effusions of unexplored causality. Their characteristic pitch-black coloring and striking first appearance upon thoracocentesis make them a bewildering occurrence even for seasoned physicians. Forming a free-from-error diagnostic work-up can be arduous and largely depends on thorough history-taking, deliberate imaging studies, and the correct biochemical profile. The upcoming article aims to raise awareness of this pathology by presenting our experience with a BPE after an episode of acute-on-chronic (ACP) pancreatitis and the confounding route to achieving the correct diagnosis and forming the precise therapeutic approach to this scenario. Keeping in mind that this is not a common clinical case, we strive to dispel some misconceptions and thus avoid any subsequent complications and delays in diagnosis when treating this type of effusions and their underlying pathology.

## Introduction

The pleura is a thin serous membrane that lines the inner aspect of the chest wall and the outer surface of the lung. This sheet pleats back on itself, forming the pleural cavity with its visceral and parietal layers. Normally, the pleural cavity holds somewhere around 8.5 ml of pleural fluid, improving overall lubrication and decreasing friction between the two pleural layers during the act of breathing [1]. As a rule, the visceral pleura secretes fluid, which is then absorbed by the abundant lymphatic network, comprising the framework of the parietal pleura. Any changes in either the morphological architecture of the pleural lining or incipient pressure spikes inside the vessels within this structure disrupt the sustainability of this system and cause more fluid to be produced than can be absorbed, thus resulting in the accumulation of fluid and an effusion. Adhering to the well-established Light criteria, pleural effusions are classified as either exudative or transudative based on their protein and lactate dehydrogenase (LDH) content [2]. For the purposes of this study, we have taken this a step further and have done a thorough biochemical analysis of the pleural fluid, evaluating the levels of amylase and lipase and pleural pH. Depending on their etiopathogenesis, effusions can be subdivided into being caused by malignancy, congestive heart failure, renal and liver failure, infection, and effusions due to pulmonary embolism. Despite being less common, attention should also be given to traumatic and tuberculous pleural effusions, effusions due to serositis, and effusions due to pancreaticopleural and cerebrospinal fluid (CSF) fistulae [3].

## Case presentation

**Figure 1:**
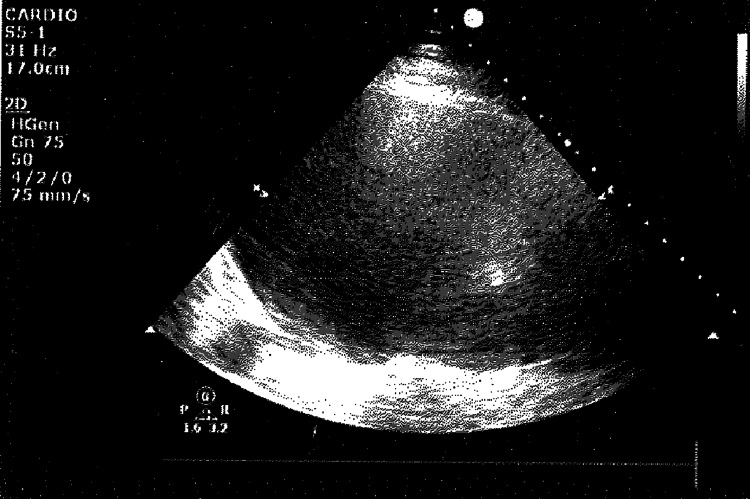
The left-sided black pleural effusion as seen on ultrasound prior to drainage

**Figure 2:**
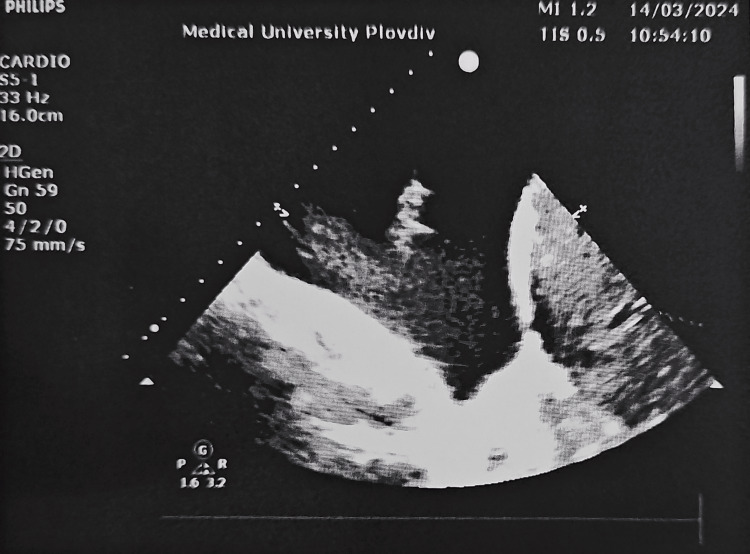
The ultrasound image of the smaller pleural effusion on the right side

**Figure 3:**
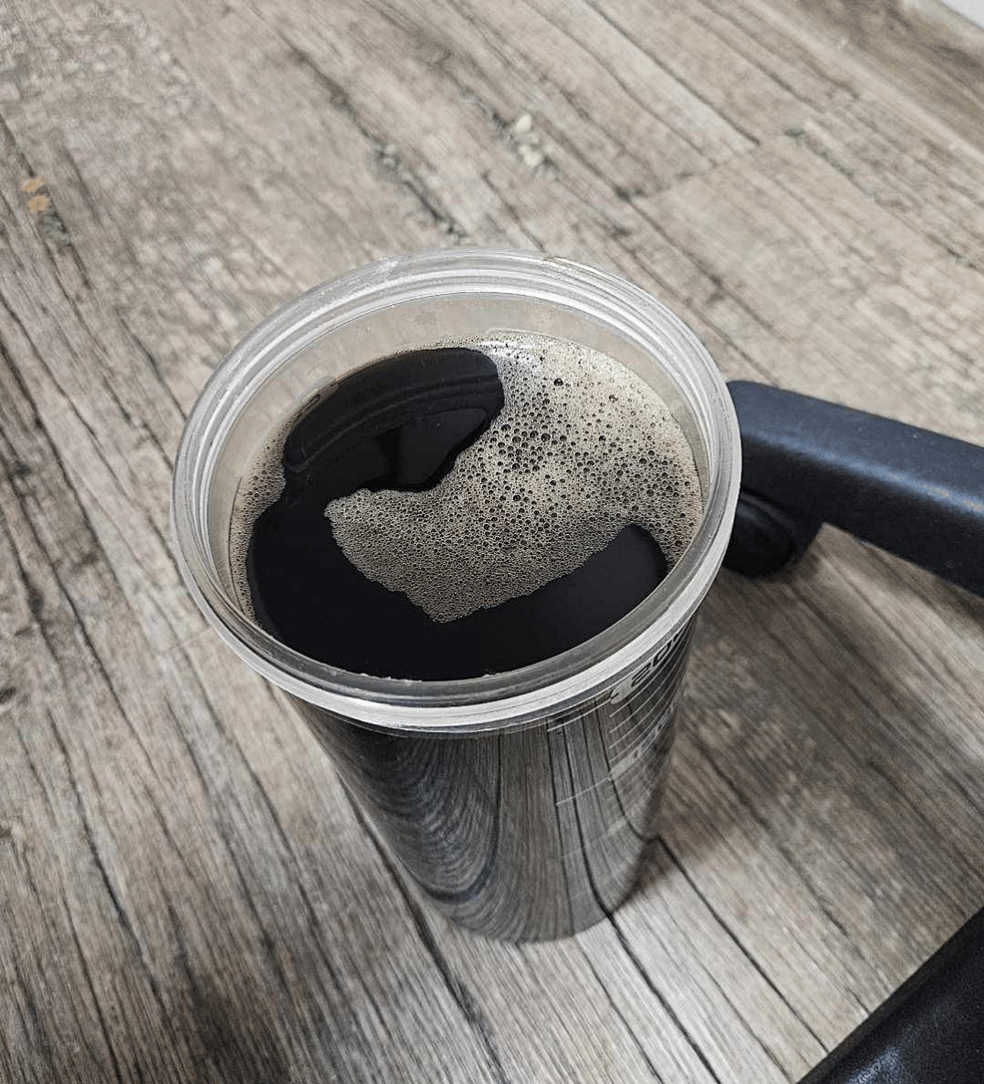
The macroscopic appearance of the fluid after evacuation

**Figure 4:**
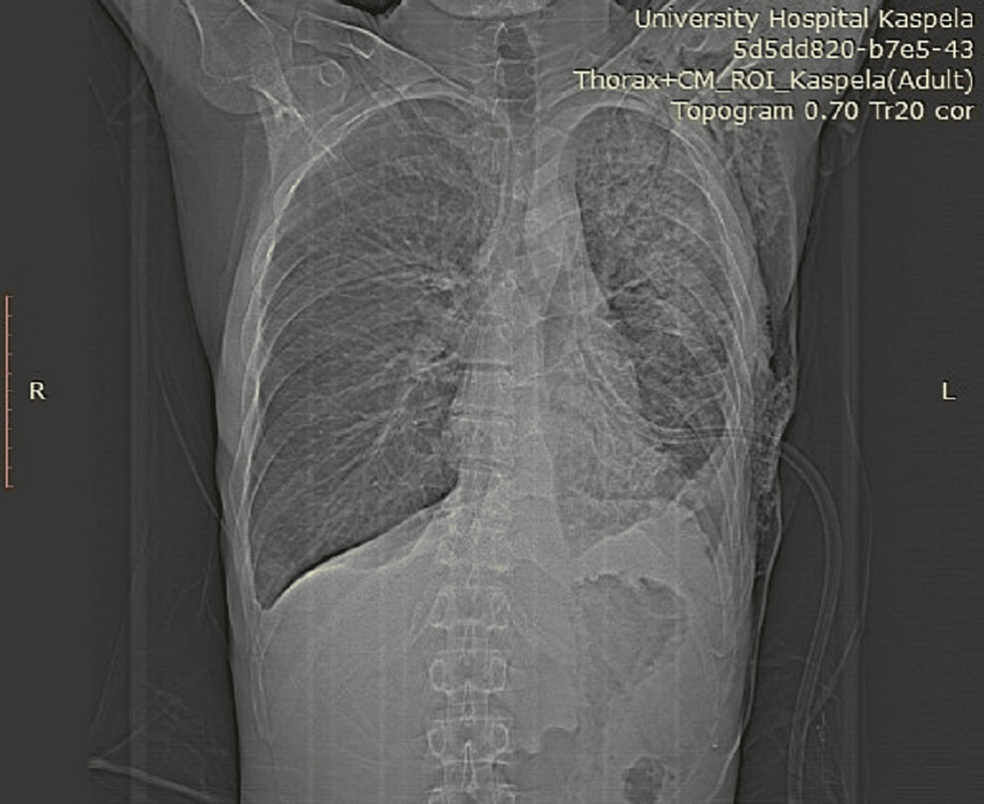
The CT just after a thoracostomy tube is placed, demonstrating a hydropneumothorax on the left

**Figure 5:**
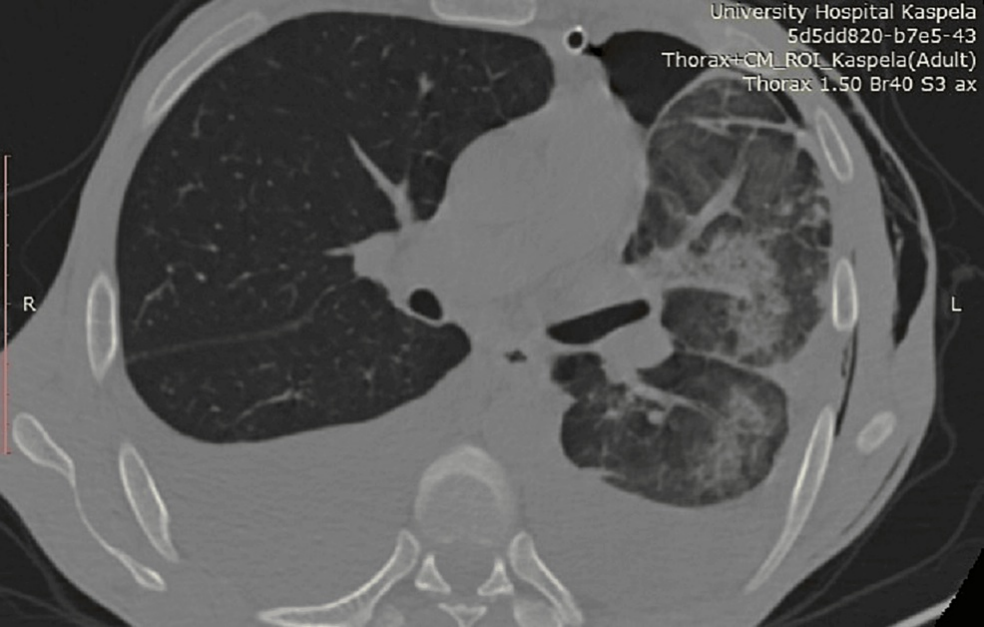
An axial view of the CT just after a thoracostomy tube is placed, noting the bilateral effusions

**Figure 6:**
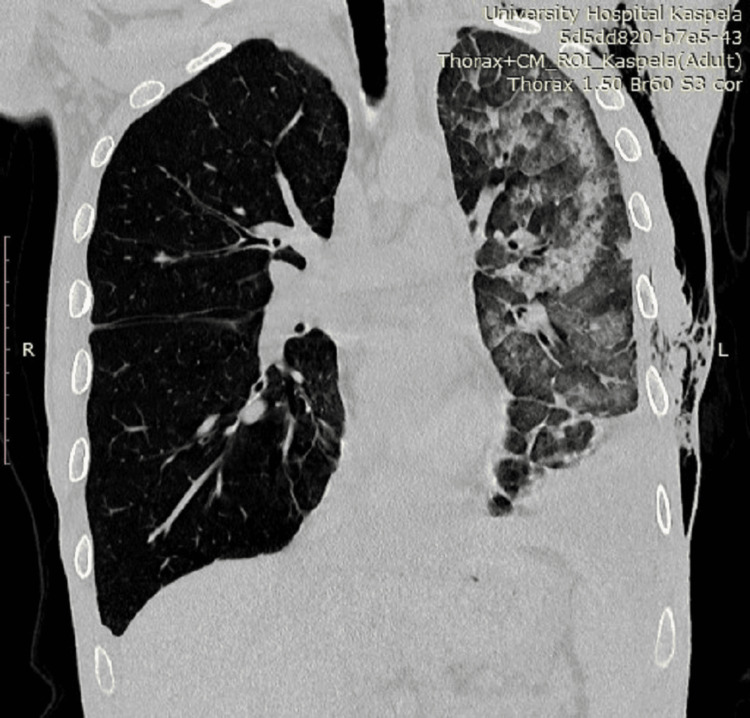
The infiltrative lesion in the parenchyma of the left lung, visualized on CT, as well as subcutaneous emphysema on the left side of the chest, a result of the carried out chest drainage

**Figure 7:**
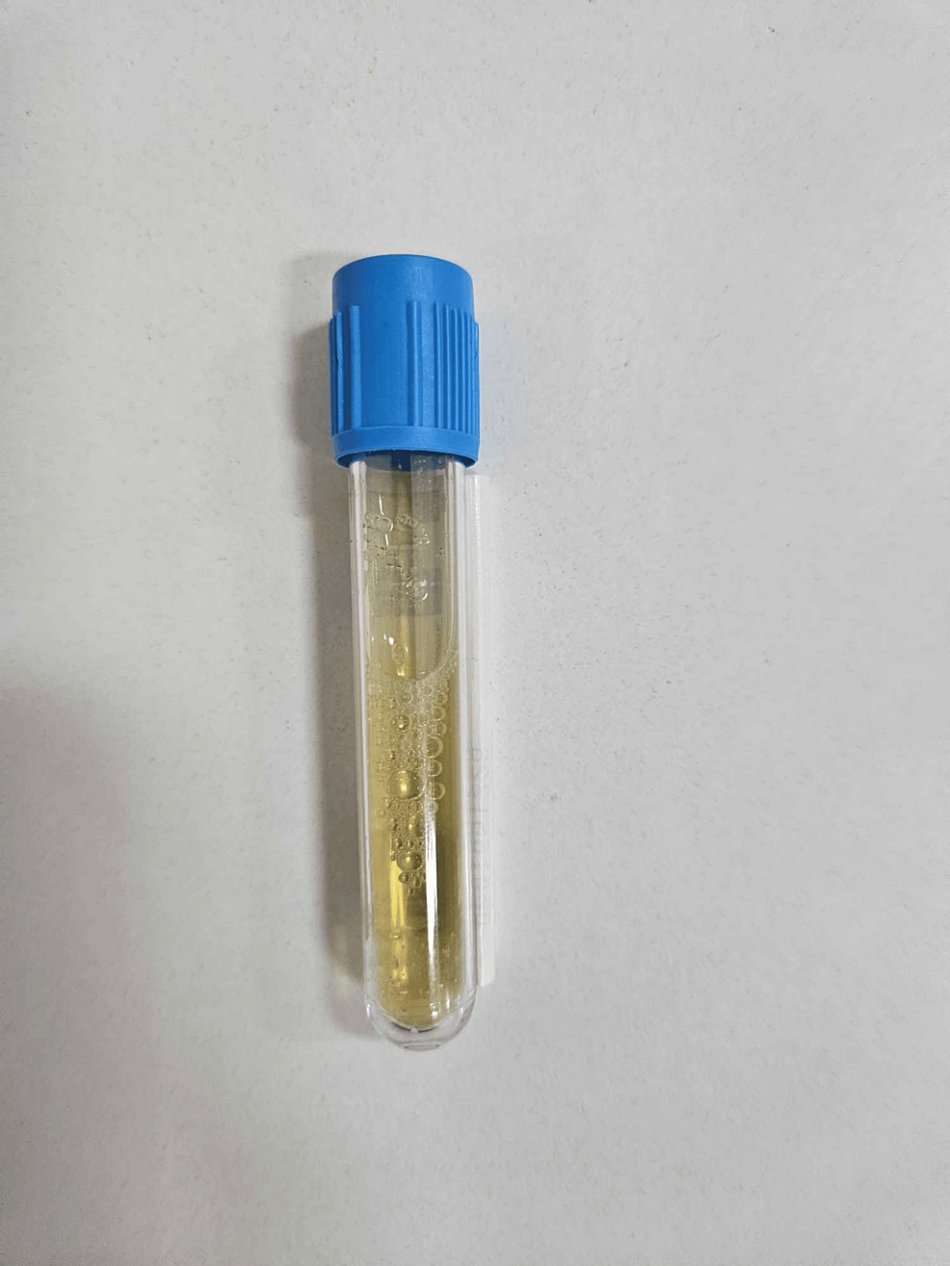
The noticeable difference in color of the pleural fluid, evacuated from the right pleural cavity

**Figure 8:**
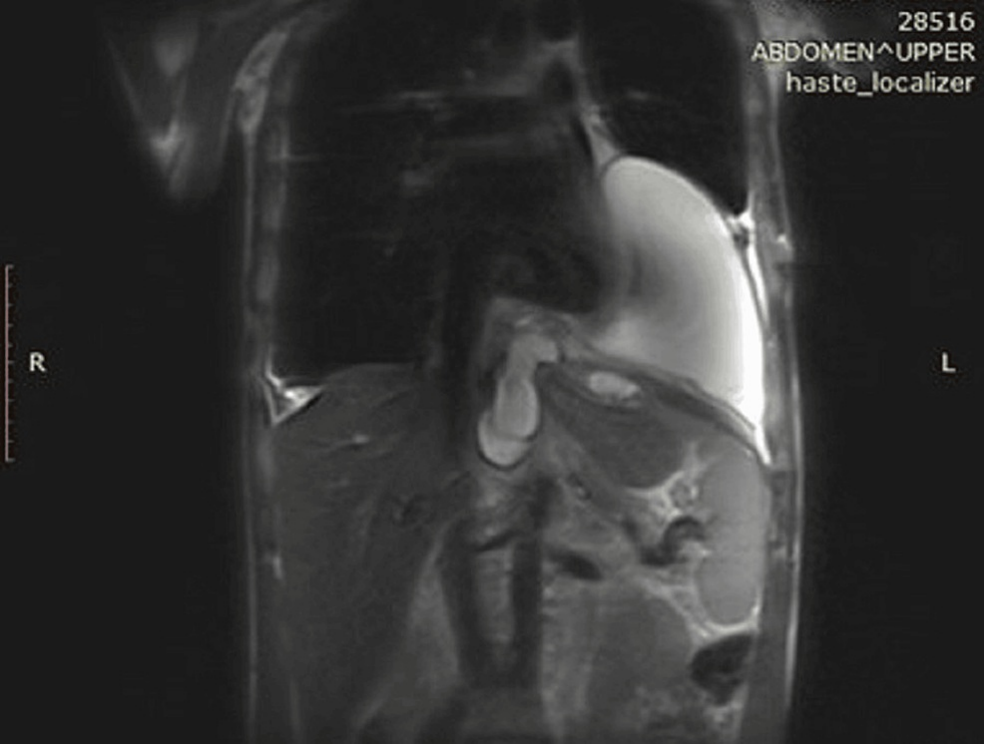
The pancreatic cysts, invading the mediastinum and left pleural cavity as seen on MRI

A 40-year-old male was admitted to the Clinic of Thoracic Surgery with complaints of severe dyspnea, which had worsened in the span of a week to an extent where it was now impossible for him to lie down, a persistent cough, and chest pain. He disclosed a history of alcohol abuse, and medical documentation revealed a recent episode of acute-on-chronic (ACP) pancreatitis. It was then that he was informed he had a 10-cm pancreatic cyst, measured via ultrasound, which he had to follow up on after treatment. Upon examination, there was a dull sound of percussion on both sides of the chest correlating with faint to absent breathing on auscultation. An ultrasound examination revealed a massive pleural effusion on the left side (Figure 1) and a large effusion on the right (Figure 2). An emergency thoracentesis was carried out, during which we evacuated 2 l of intensely black pleural fluid (Figure 3). In the following days, as the patient's condition improved after draining an additional 2 l on the same side, respiratory symptoms resolved, and we decided to insert a chest tube in order to fully empty the pleural cavity and expand the lung. A CT scan was done shortly after draining a total of 7 l of black pleural fluid. It showed a collapsed lung with heavy infiltrative lesions in the parenchyma of the left lung, as well as a right-sided pleural effusion (Figure 4, Figure 5, and Figure 6). A pleural tap was done on the right side, evacuating 1.5 l of clear pleural fluid (Figure 7). Extended biochemical analysis of the pleural fluid revealed nothing of relevance; however, the black pleural fluid came back as an exudate with extreme levels of amylase and lipase and tested positive for coagulase-negative *Staphylococcus*. Alpha-amylase in the pleural fluid was 15860 U/L, while lipase in the pleural fluid was 35980 U/L. The patient was started on specific antibiotic medications, and a follow-up microbiological study on the fifth postoperative day showed no pathological growth. The control chest X-ray demonstrated a fully expanded lung, which allowed us to remove the drain on the seventh postoperative day. He was then transferred to the Clinic of Gastroenterology in order to determine the root cause of the black pleural effusion (BPE). An abdominal ultrasound and an MRI were performed, both concluding the presence of a large pancreatic cyst, which had shrunk in size as opposed to the first ultrasound study (Figure 8). Thus, the diagnosis of a pancreaticopleural fistula was suspected. An endoscopic retrograde cholangiopancreatography (ERCP) was performed. However, due to the difficult anatomy of the biliary tree, cannulation of the pancreatic duct was next to impossible. Therefore, we decided to drain the cyst into the stomach by placing a nasocystic drain. The procedure was carried out without any complications, and the patient was discharged on the second day after drainage.

## Discussion

There exists a rich color palette to visually describe the different groups of pleural effusions. Even though their hue and intensity may vary, we have most commonly come across the typical serous, pale yellow, and blood-tinged, reddish pleural effusions. This makes the appearance of intensely dark fluid, sometimes colloquially referred to as a soy sauce effusion in the pleural cavity, an uncommon sight. Therefore, we decided to go through the available scientific literature on the matter, discovering that based on the underlying cause, all cases can be classified into four categories [4]: (1) infection: associated with *Aspergillus niger*/*Rhizopus oryzae*; (2) malignancy: most commonly malignant melanoma and not so often adenocarcinoma of the lung and mediastinal cystic teratoma; (3) hemorrhage and hemolysis: in cases of non-small cell lung cancer or a rupture of a pancreatic pseudocyst; and (4) other causes: described cases include charcoal-containing empyema, Boerhaave syndrome, crack cocaine, thoracic endometriosis, rheumatoid pleurisy, and traumatic esophageal rupture. Furthermore, a study by Del Prado-Rico et al. revealed that from the years 1950 to 2023, there were a total of 38 reported cases of BPEs. Twelve were due to malignancy. Fifteen were reported to be secondary to a pancreatic pseudocyst with a pancreaticopleural fistula. Three were a result of infection. There were also isolated cases listing crack cocaine, rheumatoid pleurisy, a bronchopulmonary fistula, and Boerhaave hydropneumothorax as possible culprits [5]. The diagnosis of a pleural effusion always starts with the physical examination itself. Even if we omit the advanced imaging studies, a dulled sound on percussion with absent breathing on one side is highly indicative of an effusion. Radiography and CT further complement a detailed physical. Nevertheless, ultrasonography proves to be the gold standard when it comes to this pathology with a recent meta-analysis showing a sensitivity of 94% and a specificity of 98%, placing it above all other imaging methods [6]. A diagnostic thoracentesis under local anesthesia, after pinpointing the maximum distance of the effusion via ultrasound, is the most straightforward way to obtain fluid for analysis. Routinely, it is advised for a pleural effusion to be studied for protein, LDH, comparing their levels to those in the blood as per the aforementioned Light criteria: pH, albumin, amylase, lipase, triglycerides, cholesterol, tumor markers, and cell 8 of 9 count [7]. Some cases of BPE are known to have resolved after chest drain thoracentesis alone; however, an occult condition should always be suspected. A pancreaticopleural fistula warrants an ERCP to be carried out. If a fistula is confirmed, a stent could be placed to block the leakage of fluid into the pleural cavity. When infection is concerned, antibiotic therapy in accordance with a microbiological study of the pleural fluid is recommended. In pleural carcinomatosis, fine powder talc pleurodesis is the method of choice to prevent recurrence. Other causes should be approached on a case-by-case basis with no standardized management having been described.

## Conclusions

The wide array of pleural effusions makes this pathology a major diagnostic and therapeutic difficulty. BPEs pose a perplexing conundrum which almost always masks a hidden condition. Adopting a cautious approach, diagnosis should always involve extended biochemical analysis, tailored to each specific situation. Some cases can be resolved with chest tube drainage on its own. However, in certain situations, a multidisciplinary team should be called to action so as to determine the best therapeutic plan.
